# Uterus didelphys: the first case report on molecular profiling of endometrial tissue from both uterine cavities

**DOI:** 10.1186/s12958-024-01330-7

**Published:** 2025-01-04

**Authors:** Alberto Sola-Leyva, Bárbara Romero, Analuce Canha-Gouveia, Inmaculada Pérez-Prieto, Nerea M. Molina, Eva Vargas, Juan Mozas-Moreno, Clara Chamorro, Merli Saare, Andres Salumets, Signe Altmäe

**Affiliations:** 1 Department of Gynecology and Reproductive Medicine, Karolinska University Hospital, Huddinge, Stockholm, 14183 Sweden; 2 Division of Obstetrics and Gynecology, Department of Clinical Science, Intervention and Technology, Karolinska Institutet, Huddinge, Stockholm, 14183 Sweden; 3 Celvia CC, Competence Centre on Health Technologies, Tartu, 50411 Estonia; 4 Reproduction Unit, UGC Obstetrics and Gynaecology, University Hospital Virgen de las Nieves, Granada, 18014 Spain; 5 Instituto de Investigación Biosanitaria ibs.GRANADA, Granada, 18012 Spain; 6 Department of Physiology, Faculty of Veterinary, University of Murcia, Murcia, 30100 Spain; 7 Department of Biochemistry and Molecular Biology I, Faculty of Sciences, University of Granada, Granada, 18071 Spain; 8 Systems Biology Unit, Department of Experimental Biology, Faculty of Experimental Sciences, University of Jaén, Jaén, 23071 Spain; 9 Department of Obstetrics and Gynaecology, Faculty of Medicine, University of Granada, Granada, 18071 Spain; 10 Consortium for Biomedical Research in Epidemiology & Public Health (CIBER Epidemiología y Salud Pública-CIBERESP), Madrid, 28029 Spain; 11 Unidad Provincial de Anatomía Patológica, Hospital Virgen de las Nieves, Granada, 18014 Spain; 12 Department of Obstetrics and Gynaecology, Institute of Clinical Medicine, University of Tartu, Tartu, 51014 Estonia

**Keywords:** Uterus didelphys, Microbiome, Metabolome, Chronic endometritis, Endometrial receptivity

## Abstract

**Background:**

A didelphic uterus represents a unique and infrequent congenital condition in which a woman possesses two distinct uteri, each with its own cervix. This anomaly arises due to partial or incomplete merging of the Müllerian ducts during the developmental stages in the womb. Accounting for uterine malformations, a didelphic uterus is a relatively rare condition, affecting approximately 0.5–2% of the population and is considered one of the more uncommon types of uterine abnormalities.

**Methods:**

This case report aims to study the physical separation in uterine didelphys and its impact on endometrial microbiome and inflammation, and the patterns of endometrial receptivity observed.

**Results:**

Endometrial receptivity analyses revealed a similar receptive state in both uteri, both in the early receptive phase. Differential markers of chronic endometritis, including CD138, and MUM1-positive cells, were observed when comparing endometrial biopsies from both uteri. The right uterus exhibited a higher prevalence of these positive cells. Regarding the microbiome, significant differences were found between the uteri, notably in the right uterus, a clear non-dominance of lactobacilli and the presence of genera such as *Staphylococcus*, *Streptococcus*, and *Acinetobacter*. Additionally, the right uterus presented a less ‘favourable’ microenvironment, a characteristic that was also reflected in the right cervix; both sites presenting less lactobacilli than the left side samples. A distinct metabolomic signature associated with the physical separation of the uteri contributed to the differences in endometrial milieu.

**Conclusions:**

Our study revealed that physical separation, among other factors in uterus didelphys, affects the endometrial microbiome, metabolome, and inflammatory state, with significant microbiome variation observed between the uteri, although similar endometrial receptivity patterns were noted.

**Supplementary Information:**

The online version contains supplementary material available at 10.1186/s12958-024-01330-7.

## Background

Uterus didelphys, often referred to as double uterus, represents a unique and infrequent congenital condition in which a woman possesses two distinct adjacent uteri, each with its own cervix, and is commonly associated with a longitudinal vaginal septum in up to 30% of the cases [1]. This anomaly arises due to partial or incomplete merging of the Müllerian ducts during the developmental stages in the womb. Normally, these ducts would fully combine to create a single uterus, but in the case of uterus didelphys, the fusion is incomplete, leading to the formation of two separate uterine structures [2]. Accounting for uterine malformations, a uterus didelphys is a relatively rare condition, affecting approximately 0.5–2% of the population [3]. Women with uterus didelphys exhibit a lower clinical pregnancy rate, lower live-birth rate, and a higher rate of first-trimester pregnancy loss [4]. Given its low prevalence, routine screening is not indicated, and studies on this anomaly are scarce. Therefore, individual case assessment and personalized management are crucial.

Beyond the direct effects of uterine anatomical anomaly and more intricate physiological features associated with uterine malformations, various other factors, including molecular alterations and microenvironment, play their roles in determining the functionality of the uterus, in fertility and infertility. The inner lining of the uterus, i.e., endometrium is a highly dynamic tissue that changes in response to hormones to promote the embryo implantation and pregnancy development. Determining the receptivity of the endometrium, the window of embryo implantation, has been a significant challenge over recent decades, evolving from histological evaluation to transcriptome studies [5, 6]. The use of both microarray and RNA-sequencing based techniques has provided a wealth of information, and currently, different add-on molecular tests are available [7]. While there is insufficient data to recommend the routine use of any commercially available tests of endometrial receptivity to diagnose the cause of recurrent implantation failure, one of the most severe forms of infertility, assessment of specific aspects of endometrial function by testing can be considered [8]. Nevertheless, these molecular endometrial receptivity tests serve as valuable tools for precisely dating the menstrual cycle phases of endometrial samples through transcriptome profiling [9]. Moreover, advancements in all omics profiling technologies, extending from DNA to metabolites have significantly contributed to improved understanding of the molecular markers associated with the receptive mid-secretory endometrial function in fertility and infertility-associated diseases [10–15]. The studies of microbes in the female reproductive tract have introduced an additional layer of knowledge, along with increased complexity, challenges, and controversies. In general, the microbial diversity, i.e., species richness gradually increases from the lower (vagina and cervix) to the upper female reproductive tract (uterus, Fallopian tubes and ovaries) [16–18]. The presence of specific microorganisms, as well as the lactobacillus dominance or non-dominance within the female reproductive system has been associated with various pathologies and can significantly impact reproductive success [19, 20]. In particular, the condition of chronic endometritis (CE), marked by the invasion of immune cells into the endometrium, has been associated with a specific microbial signature in the endometrium which is related to female poor reproductive outcomes [21–23].

In this article, we delineate the immunological traits, metabolomic, transcriptomic, and microbiomic profiles individually for endometrial tissue samples obtained from both uterine cavities of a woman experiencing infertility with uterus didelphys.

## Case report

**Fig. 1:**
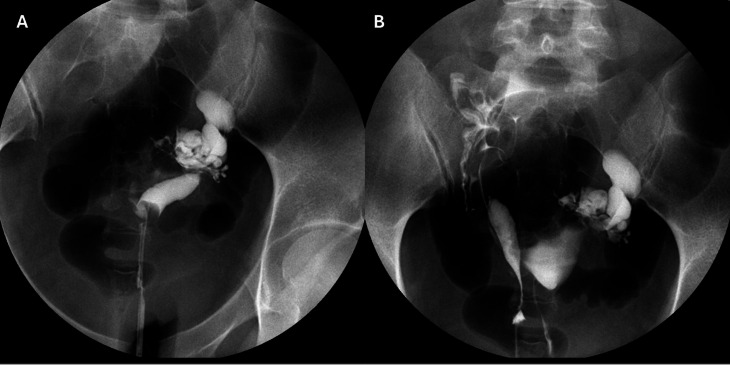
Hysterosalpingogram confirmation of uterus didelphys. **A.** Cannulation of the external cervical orifice of the left cervix and iodinated contrast revealing filling of the endometrial cavity with no filling defects. The dilation of the ampullary portion of the left fallopian tube, indicative of a non-permeable left hydrosalpinx, is also shown. **B.** Cannulation of the external cervical orifice of the right cervix and the filling of the endometrial cavity without defects and a normal calibre and appearance of the right fallopian tube with contrast exiting into the peritoneal cavity

In September 2020, a 36-year-old woman was referred to the Reproduction Unit of the Virgen de las Nieves University Hospital of Granada (Spain) due to primary infertility following two years of unprotected sexual intercourse. Patient consent was provided for publication of this report. Her clinical anamnesis includes a family history of endometrial and breast cancer, but no reported cases of infertility. She was a non-smoker with a BMI of 31.5 kg/m². Hormonal analysis revealed a low ovarian reserve (AMH 0.37 ng/mL). Upon vaginal examination, two cervixes of normal size were visualized, separated by a septum in the upper vaginal third. By transvaginal ultrasound, two uteri of normal size were visualized, with normal endometria and ovaries without pathology with a low antral follicle count. The hysterosalpingogram confirmed the uterus didelphys (Fig. 1). The external cervical orifice of the left cervix was cannulated by introducing the catheter into the homolateral uterine cavity and injecting 12 ml of water-soluble iodinated contrast. Filling of the endometrial cavity was observed, which shows no filling defects (Fig. 1A). The left fallopian tube presented dilation of the ampullary portion, with retention of the contrast material, without evidence of passage into the peritoneum, consistent with a non-permeable left hydrosalpinx. Secondly, the external cervical orifice of the right cervix was cannulated, with an injection of 8 ml of water-soluble iodinated contrast (Fig. 1B). Filling of the endometrial cavity was observed, showing no filling defects. The right fallopian tube displayed a normal calibre and appearance, demonstrating the exit of the contrast into the peritoneal cavity (Fig. 1B). A 17 mm cyst with blood-filled content in the right adnexa, suggesting the presence of an endometrioma, was identified using nuclear magnetic resonance imaging (MRI).

### Sample collection

All the samples to characterize the microbiome, metabolome, transcriptome, and immunological features of the uterus didelphys of the recruited patient were collected during the mid-secretory phase of the menstrual cycle (LH +7), measured by LH strips (Clearblue^®^). For the microbiome analysis, a vaginal swab (V) (eNAT^®^ 606CS01R; COPAN ITALIA, Brescia) and two cervical swabs (eNAT^®^ 606CS01L) (Left cervix, CL; and right cervix, CR) were collected. The collection of endometrial samples from both uterine cavities (Left uterus, UL; right uterus, UR) was carried out using the Tao Brush IUMC endometrial sampler (Cook Medical, Madrid, Spain). To ensure minimal contamination with bacteria from the lower reproductive tract, Tao Brush IUMC was carefully closed within the uterine cavity after sample collection. Subsequently, the samples from the brush were stored in Copan eNAT^®^ transport system (eNAT^®^ 606 C) and stored at a temperature of -80°C.

Subsequently, to study the transcriptome, metabolome, and immunological features of the double uterus, an endometrial biopsy was obtained from both sides (UL; UR) using an endometrial curette device (Gynétics Medical Products, Hamont-Achel, Belgium). The collected tissue was placed into a sterile tube and was divided into three portions. One of them was designated for Pathological Anatomy Service of the hospital to screen for the endometrial pathologies, and to determine the menstrual cycle phase and the presence of CE. Briefly, the first part was fixed in 10% neutral-buffered formalin for conventional histology using haematoxylin-eosin and CD138 (ref. MAD-000735QD-3/V) / MUM1 (ref. MAD-000470QD-3/D) staining following manufacturer recommendation (Vitro Master Diagnostica, Spain). Biopsies were assessed by two pathologists specialized in histological endometrial analysis. The next tissue sample was stored in RNALater for transcriptomic characterization of the endometrial status, and the third biopsy piece was snap-frozen for metabolome analysis. All samples were stored at -80ºC for further analyses.

### Endometrial dating and determining chronic endometritis

**Fig. 2:**
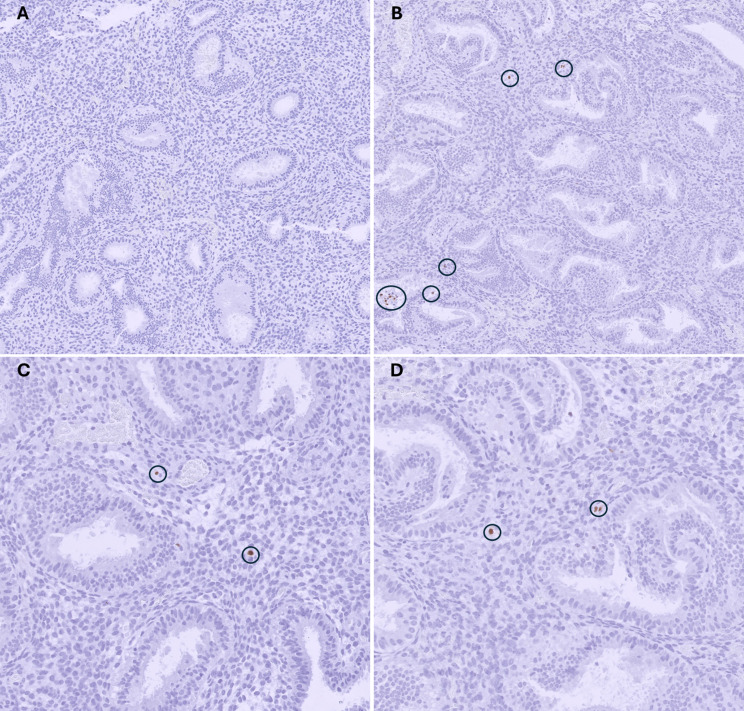
Histology of the endometrium from both uteri, left (**A**) and right (**B**, **C** and **D**) stained with MUM1 in immunohistology staining. MUM1-positive cells are indicated by black rings. **A** and **B** original magnification X10. **C** and **D** original magnification x20

The histological dating of the endometrium was performed according to the Noyes’ criteria [24]. Both uteri were dated as being in the mid-secretory phase, corresponding to the sampling day, i.e., the cycle day 21. CE was diagnosed through the identification of 1 or more CD138 and/or MUM1 positive plasma cells per 10 high-power field examined in immunohistology staining. Notably, CD138 staining was negative in both endometrial samples (data not presented). The pathologists confirmed the presence of CE in the right-sided uterus, demonstrating MUM1-positive staining for plasma cells, as depicted in Fig. 2B, C and D. Conversely, no MUM1 positive cells were found in the left-sided uterus (Fig. 2A). The concurrent use of CD138 and MUM1 staining mitigates the risk of underestimating the CE diagnosis inherent to single-staining approaches, offering a more reliable assessment of CE [25].

### Endometrial receptivity assayed by transcriptomic profile

Total RNA of endometrial tissue samples was extracted using miRNeasy Micro kit (Qiagen, Hilden, Germany) followed by RiboZero kit (Qiagen, Venlo, Netherlands) processing to remove rRNA. The Stranded Total RNA Prep technology (Illumina, USA) was used to generate the libraries. Equimolar libraries were pooled and sequenced using the S2 flow cell, paired-end 100 bp on a NovaSeq 6000 sequencer (Illumina, San Diego, CA, USA). Gene expression profiling of 72 genes analysed with 57 endometrial receptivity-associated biomarkers [14], 11 additional genes relevant to window of implantation (Supplementary Table S1), and four housekeeper genes was estimated using the beREADY algorithm (www.beready.ee, Celvia CC, Competence Centre on Health Technologies, Tartu, Estonia) [26] to confirm the receptivity status of endometrial samples. The results of the beREADY test showed that both endometrial samples, and therefore both uteri, represent early/mid-receptive phase, confirming the endometrial histology dating and demonstrating no differences in endometrial receptivity status between the left and right uteri.

### Microbiome analysis

**Fig. 3:**
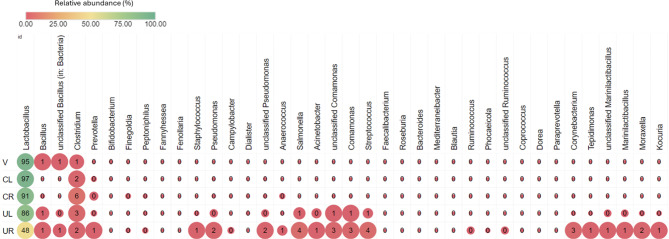
Heatmap of bacteria relative abundance from vagina (V), left cervix (CL), right cervix (CR), left uterus (UL) and right uterus (UR). The maximum size of the circles indicates a relative microbial abundance grater than 1%

The microbiome of both uterine samples, both cervical samples and vagina were profiled by amplifying the bacterial-specific V4 hypervariable region of the 16S rRNA gene and sequencing. Briefly, DNA was extracted by using the DNA extraction kit (Qiagen QIAamp UCP with Pathogen Lysis Tube S). The primers used were 515F (5’-GTGYCAGCMGCCGCGGTAA) and 806R (5’- GGACTACNVGGGTWTCTAAT). The bioinformatic analysis was performed by using Kraken2 [27]. Microbiome diversity analyses were also conducted under RStudio (R version 4.3.2 (2023-10-31 ucrt)) using phyloseq, vegan, microViz, and ggplot2 R packages [28–30]. The relative microbial abundances for the different body sites (i.e., vagina, both cervixes and uteri) are shown in Fig. 3. The vaginal and cervical microbiomes were characterised by clear dominance of lactobacilli. Slight differences in lactobacilli abundance were found between the two cervixes (CL = 96.6% vs. CR = 90.7%). However, in terms of endometrial microbiome, different microbial compositions were noted (Fig. 3). Concretely, the endometrial microbiome from the right was not dominated by lactobacilli (48.2%), but with other microbial genera like *Streptococcus*, *Staphylococcus*, *Bacillus* and *Streptococcus* comprising a larger percentage of the microbial composition (Fig. 3). These microbial taxa present in UR have been linked to endometrial dysfunction, and particularly to the CE [31, 32]. What is interesting is that while UR presented less ‘favourable’ microenvironment, also same was reflected in the right cervix, both sites presenting less lactobacilli than the left side samples.

The alpha diversity was evaluated by the Shannon, Simpson and Chao1 indexes. As outlined in Table 1, all values indicate that the least diverse niche was the vagina and cervix, followed by the uterus. The right uterus and cervix displayed higher alpha diversity indexes compared to the left, which is consistent with the observed decrease in lactobacilli abundance in the sites of the right cervix/uterus. The diversity indices indicate that ‘unfavourable’ uterine microenvironment is already detected at the cervical sample level. Also, as previously demonstrated, the diversity increased when ascending from the vagina and cervix to the uterus [16].

**Table 1:** Estimation of alpha diversity indexes of bacterial population

Samples	Shannon	Simpson	Chao1
Vagina (V)	0.28	0.09	33
Left cervix (CL)	0.20	0.07	16
Right cervix (CR)	0.47	0.17	42
Left uterus (UL)	0.85	0.25	71
Right uterus (UR)	2.68	0.76	107

**Fig. 4:**
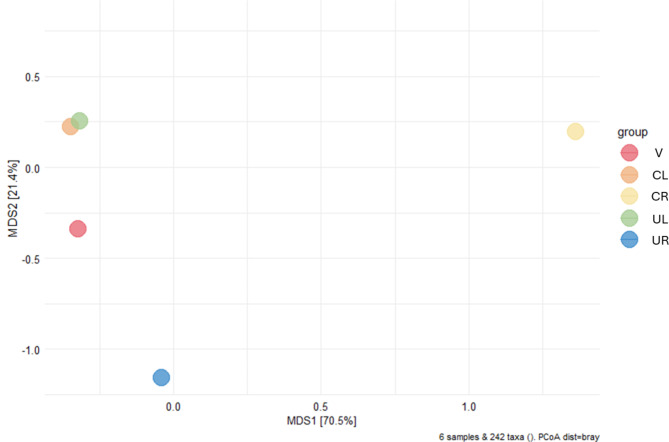
Principal coordinate analysis (PCoA) plot based on Bray-Curtis dissimilarity. Dots are positioned according to the distances between microbial communities in the different samples: vagina (V), left cervix (CL), right cervix (CR), left uterus (UL), and right uterus (UR)

A principal coordinate analysis (PCoA), based on Bray-Curtis distances measured the dissimilarity of microbial community compositions across various sample sites (Fig. 4). The first principal coordinate (MDS1) accounts for 70.5% of the variation. In contrast, the second principal coordinate (MDS2), explaining 21.4% of the variation, does not clearly differentiate between the remaining sites V, CL, CR, UL, and UR. Despite these findings, UR together with CR samples appears to be positioned further from the central cluster of the other female reproductive tract samples (V, CL, UL), suggesting that the microbial community composition of the UR and CR samples are distinct compared to the other sites within the female reproductive tract. The UR and CR samples show a noticeable deviation from the other reproductive tract sites, particularly compared to its contralateral UL/CL samples. This finding reflects a clear lateral asymmetry within the microbial communities between the two uterine cavities of the patient’s uterus didelphys with an effect also on the cervical microenvironment.

### Metabolomic profile

The untargeted metabolomics analysis of both endometrial biopsies (UR and UL) was conducted at Metabolon Inc., Morrisville, NC, USA. This analysis utilized a system consisting of four separate ultrahigh-performance liquid chromatography-tandem mass spectrometry (UPLC–MS/MS) instruments, as previously described [33, 34]. A total of eight hundred sixty-four metabolites from diverse chemical classes were identified from both endometrial tissue samples using the untargeted metabolomic approach. These metabolites encompassed amino acids, lipids, nucleotides, carbohydrates, and xenobiotics, among others. The list of identified compounds is provided in Supplementary Table S2.

**Fig. 5:**
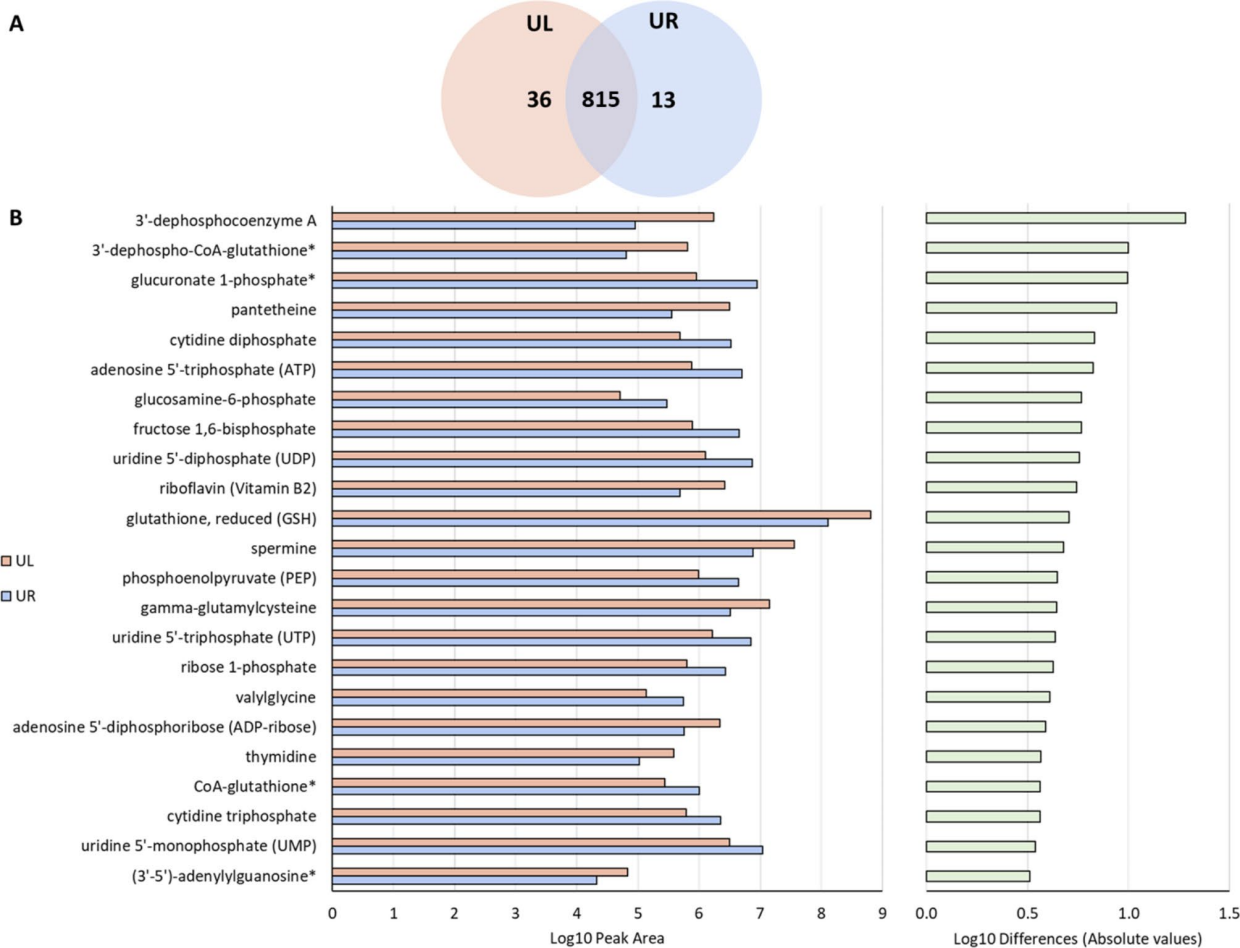
Metabolomic profile of uterus didelphys.** A**. Venn diagram representing the numbers of metabolites identified in endometrial samples (Left uterus, UL; right uterus, UR). **B**. Bar chart illustrating the twenty metabolites with the greatest differences (absolute values) in log peak area detected between the samples (blue for right uterus (UR), orange for left uterus (UL)). The right panel evidences the Log10 differences in peak areas in absolutes values among UR and UL. Bars indicate the magnitude of differences for each metabolite.

Our comprehensive analysis revealed that both uteri primarily share the same metabolomic profile, with 815 common metabolites detected in both uteri (Fig. 5A). However, variations in the peak areas corresponding to individual compounds were observed between the samples (Fig. 5B). Among the metabolites exhibiting the most significant differences between both uteri were metabolites representing coenzyme A and glutathione metabolism, including 3’-dephosphocoenzyme A and 3’-dephospho-CoA-glutathione. Coenzyme A is implicated in various metabolic pathways, such as fatty acid metabolism and the citric acid cycle [35]. CoA-glutathione likely participates in cellular detoxification processes, which are crucial for maintaining cellular homeostasis, particularly in the uterus [36].

**Fig. 6:**
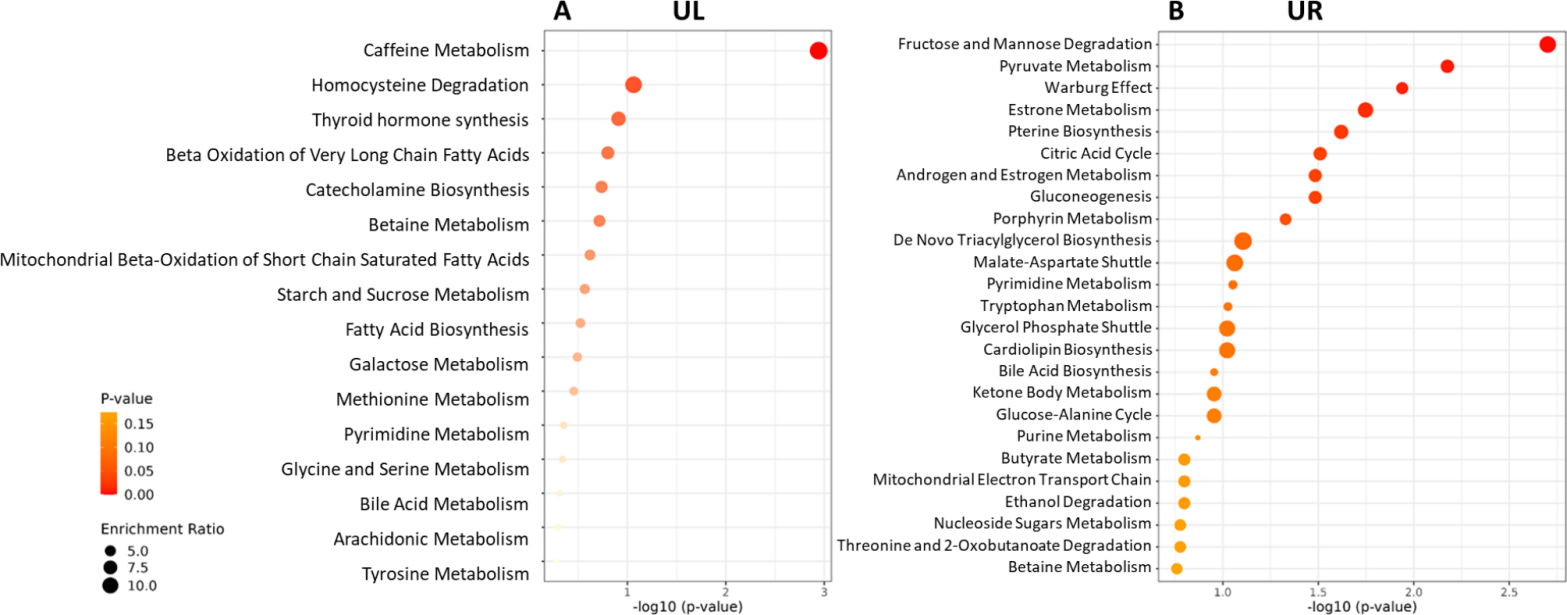
The enriched metabolic pathways in the left (**A**) and right (**B**) uterus based on the group of metabolites that were uniquely detected in each uterus. This dotplot chart was done using the MetaboAnalyst 6.0 tool  (https://www.metaboanalyst.ca). The enrichment ratio was calculated as the number of hits within a particular metabolic pathway divided by the expected number of hits. P-values were obtained from the Student’s t test univariate analysis after linear regression and pareto scaling normalization

It is noteworthy to highlight that several key metabolites were exclusively detected in one sample (Fig. 5A), specifically 13 in the right uterus and 36 in the left uterus (Supplementary Table S2). These metabolites were involved in different biochemical pathways, potentially reflecting different uterine microenvironments (Fig. 6).

## Discussion

Women with uterus didelphys experience lower clinical pregnancy rates, reduced live-birth rates, and higher rates of first-trimester pregnancy loss, highlighting the significant impact of this condition on reproductive outcomes. This case report represents a noteworthy contribution to the field by exploring critical aspects of endometrial quality, such as the microbiome, metabolome, endometrial receptivity, and associated inflammatory markers, to improve pregnancy outcomes in this under-researched pathology. Furthermore, this study introduces a novel aspect, suggesting that this uterine anomaly may confer differing prognoses for each uterine cavity, influenced by varying predisposing factors that may enhance or hinder their respective gestational capacities. The physical separation of two uterine cavities in the uterus didelphys seems to participate in the differentiation in terms of the microenvironmental composition demonstrated by immunological factor MUM1 and microbiome. The clear changes in the microbiome, with nearly 90% and < 50% abundance of lactobacilli in left- and right-sided uteri, respectively, along with differences in the cervical microbiome and the presence of CE-associated microbes in the right-sided uterus, were associated with the CE diagnosis. This diagnosis was based on the high numbers of the MUM1-positive cells on the right side of the double uterus. Despite this, endometrial receptivity status was consistent across both endometrial samples, classified as early/mid-receptive. Regarding the endometrioma, numerous studies have reported its association with endometriosis, adhesions, infertility, and diminished ovarian reserve, particularly in older patients and those with a history of ovarian surgery [37]. Additionally, there is growing evidence supporting the role of the microbiome in the development and progression of endometriosis through inflammatory pathways. Dysbiosis observed in endometriosis is increasingly recognized as both a cause and a consequence of its pathogenesis [38]. In this context, the presence of a right adnexal endometrioma, coinciding with a more pathogenic endometrial microbiome in the right uterine cavity, may represent either a causal relationship or a coincidence. Interestingly, this phenomenon was not observed on the left side, despite the presence of a chronic hydrosalpinx, which is also associated with dysbiosis [39]. These findings suggest that the observed pathology and its associated microbiome are likely dynamic rather than static conditions. This represents a paradigm shift with significant clinical implications, emphasizing the necessity of independently studying both uterine cavities when endometrial evaluation is warranted in cases of assisted reproduction. In scenarios such as embryo implantation failure in a didelphys uterus, if supported by larger studies, the possibility of independent endometrial status in each uterine cavity, as indicated by this clinical case, would necessitate separate assessments and considerations. These would include endometrial sampling and microbiological studies for each cavity, a practice that is not currently part of standard clinical protocols.

On the other hand, metabolome analysis demonstrated distinct metabolic profiles in both uteri. The left uterus, exhibiting more ‘favourable’ microenvironment, showed high levels of metabolites involved in lipid metabolism, especially in fatty acid β-oxidation (Fig. 6A). To the contrary, the right-sided uterus, with less ‘favourable’ microenvironment, presented metabolic patterns associated with carbohydrate metabolism and a shift in energy production known as the Warburg effect (Fig. 6B). The list of differentially abundant metabolites between the two uteri included nucleotides such as cytidine diphosphate, adenosine 5’-triphosphate (ATP), uridine 5’-diphosphate (UDP), uridine 5’-triphosphate (UTP), and adenosine 5’-diphosphoribose (ADP-ribose) that are involved in nucleic acid and energy metabolism, essential for cellular function in the uterus [40, 41]. Additional metabolites like glucuronate 1-phosphate, glucosamine 6-phosphate, fructose 1,6-bisphosphate, ribose 1-phosphate, and phosphoenolpyruvate serve as intermediates in various metabolic pathways critical for energy production and biosynthesis in the uterus [42]. Furthermore, metabolites such as riboflavin (Vitamin B2), gamma-glutamylcysteine, thymidine, and spermine, known for their roles in cellular metabolism and antioxidant defence, also exhibiting notable differences between the two uteri, showing higher level in the left uterus [43, 44]. Reduced glutathione (GSH), which was more prevalent in the left-side uteri, is a critical antioxidant molecule that plays a crucial role in protecting cells from oxidative stress and maintaining redox balance. This function is vital for cellular health and function in the uterus [45]. In the exploration of the unique signatures of metabolic activity from each uterus, several metabolic pathways stand out. Specifically, in the left uterus, fatty acid β-oxidation stands out as the main energy source. The fatty acid metabolism and particularly β-oxidation pathways have been shown to play an important role for oocyte and embryo development [9, 46]. Furthermore, fatty acid β-oxidation is critical for decidualization, where endometrial stromal cells differentiate into decidual cells. Decidualization is crucial to establish and maintain a pregnancy, representing one of the most essential processes within the human endometrium throughout pregnancy [47]. Several metabolites related to caffeine metabolism, homocysteine, thyroid hormone synthesis pathway, among others were revealed in the left uterus (Fig. 6A). On the other hand, right uterus exhibited pathways like carbohydrate degradation, pyruvate metabolism and the Warburg effect. This effect has been extensively described in endometriotic lesions [48, 49, 50], supported by the visualization of the endometriotic cyst within the right ovary on an MRI scan. Altogether, the results of the current case report highlight how the application of multiomic techniques in the clinical setup can advance the understanding of patient’s complex conditions such as uterus didelphys, which may potentially influence the decisions made in assisted reproduction.

### Limitations

The present study documents a unique case suggesting that, among various factors—including hydrosalpinx, sampling method, and analytical techniques—the physical separation of the two uteri in didelphic uterus condition may influence the microenvironment of the female reproductive tract. However, these results are derived from a single case and may not be generalizable across all such conditions. Furthermore, the method employed in microbiome analysis is crucial for accurate conclusions regarding the endometrial microbiome. Due to the low microbial biomass of the endometrium, findings might be influenced by contamination from adjacent parts of the female reproductive tract or external sources. Additionally, the etiology and diagnostic methods for CE remain controversial and may be influenced by changes stemming from previous intrauterine interventions.

As this is a single clinical case, several limitations must be acknowledged regarding the observed results. It is important to recognize that infertility often involves a combination of adverse factors that complicate the achievement of pregnancy. In this instance, the patient presented with a uterine malformation, left hydrosalpinx, and an endometrioma in the right ovary. These were further compounded by the presence of CE and an unfavorable microbiota in the right uterine cavity, while the left cavity remained unaffected. A potential research avenue would be to investigate whether the differing prognostic conditions for achieving pregnancy in each uterine cavity, as suggested in this case, would also be observed in cases involving only the uterine malformation without additional complicating factors. Further and more extensive investigations into the potential independence of endometrial status in the uterus didelphys are necessary to validate this hypothesis. However, for this concept to gain traction, it must first be acknowledged and integrated into the considerations of researchers and clinicians in their studies.

## Electronic Supplementary Material

Below is the link to the electronic supplementary material.

Supplementary Material 1

Supplementary Material 2

## Data Availability

No datasets were generated or analysed during the current study.
